# Supplementary material for: All-at-once RNA folding with 3D motif prediction framed by evolutionary information
Source: Nat Methods. 2025 Oct 3;22(10):2094–106. doi: 10.1038/s41592-025-02833-w (PMC12510878; doi:10.1038/s41592-025-02833-w)
Supplement: Supplementary file 3 — Source code to run CaCoFold-R3D. Data and results. [file 41592_2025_2833_MOESM3_ESM.gz › supplemental_material/Figures/Figure_1.pdf]

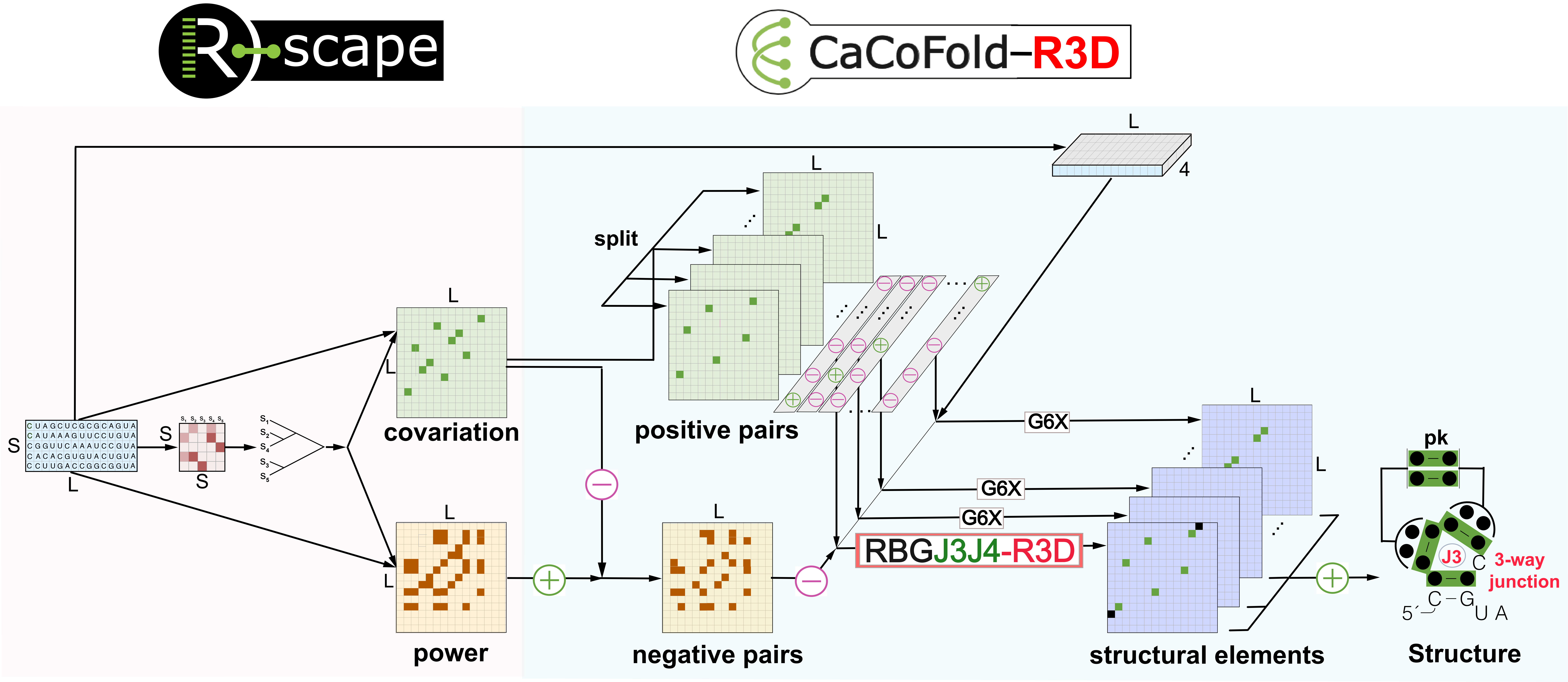
R-scape
CaCoFold-R3D
CUAGCUCGCGCAGUA
CAUAAAGUUCCUGUA
CGGUUCAAAUCCGUA
CACACGUGUACUGUA
CCUUGACCGGCGGUA
S
L
covariation
power
split
positive pairs
negative pairs
G6X
RBGJ3J4-R3D
structural elements
pk
J3
3-way junction
Structure
